# Tracing Hydrazine in Pharmaceuticals: Toxicological Concerns, Extraction Approaches, and Analytical Method Development

**DOI:** 10.1155/jamc/9609425

**Published:** 2026-10-19

**Authors:** Buhlebenkosi Mercy Donga, Rimadani Pratiwi, Aliya Nur Hasanah

**Affiliations:** ^1^ Faculty of Pharmacy University Padjadjaran Jl. Raya Bandung Sumedang KM 21 Sumedang 45363 West Java Indonesia; ^2^ Department of Pharmaceutical Analysis and Medicinal Chemistry Faculty of Pharmacy University Padjadjaran Jl. Raya Bandung Sumedang KM 21 Sumedang 45363 West Java Indonesia

**Keywords:** analytical method development, derivatization, extraction, genotoxic impurity (GTIs), hydrazine impurity, toxicology

## Abstract

Hydrazine (N_2_H_4_) is a synthetic reagent that is used in the manufacturing of various active pharmaceutical ingredients (APIs) such as isoniazid, hydralazine, and carbidopa. The major concerns associated with its presence in medications as a residual reagent or degradation product arise because of its ability to damage the genetic material, cause mutations, and potentially trigger cancer. It has been classified under Class 2 genotoxic impurity (GTI), based on the ICH M7 guideline. Its multitissue toxicities including hepatotoxicity, neurotoxicity, and hematotoxicity have led to tight regulatory limits with the EMA threshold of toxicological concern (TTC) at 1.5 µg/day. Detecting hydrazine in trace concentrations is challenging because of its low molecular weight, high polarity, absence of chromophores, and chemical instability. In order to provide a unified framework for impurity profiling, we combine hydrazine’s toxicological profiles, formation pathways, and analytical advancements. Recent advances on the extraction, derivatization, and analytical determination of hydrazine to improve its stability and detectability in pharmaceutical matrices ranging from 2020 to 2025 were collected. Comparative evaluation of the methodologies reveals that combining chemical derivatization with hyphenated techniques delivers the highest sensitivity. GC‐MS/MS stands out as the gold standard, providing exceptionally low detection limits at the sub‐ppm level with high precision and reliability, while UHPLC‐fluorescence serves as an alternative with considerable sensitivity for detecting hydrazine. Gaps in method harmonization, reference standard availability, and validation for complex formulations still remain therefore future work should focus more on standardized analytical methods, development of greener methods of derivatization, as well as use of high‐resolution detection systems.

## 1. Introduction

Pharmaceutical impurities are unintended chemical entities, foreign to the intended composition of active pharmaceutical ingredients (APIs), excipients, or solvents. As per the International Council for Harmonisation (ICH), an impurity in a new drug substance is defined as any component that is not the chemical entity itself [1]. These impurities originate from various sources, including the manufacturing process, starting materials, and degradation during storage. Their presence in APIs can carry over into final drug product, or form anew through interactions with excipients, thereby compromising purity, safety, and efficacy, and posing a direct risk to patient health if they exceed specified limits [1, 2].

According to ICH Q3A guidelines, impurities are categorized into organic, inorganic, and residual solvents [1]. Table 1 defines and summarizes the sources of organic, inorganic, and residual solvent impurities, including their examples. Among these, organic impurities are the most frequent and analytically challenging class in pharmaceutical products [3]. Unlike inorganic and residual solvents, which occur rarely and are typically known and identified [4], organic impurities can be unpredictable and may remain undetected [1, 5, 6].

**TABLE 1:** Sources of organic, inorganic, and residual solvent impurities.

Category	Description	Sources	Examples	Ref.
Organic	Unwanted organic chemical substances arising from the manufacturing process and storage of drug products	Degradation	Hydrazine, 4‐aminophenol	[1, 7, 8]
		Unreacted starting materials		
		By‐products (from incomplete reactions or unintended side reactions)		
		Intermediates		
		Residual reagents, catalysts, ligands		

Inorganic	Noncarbon‐based substances typically originating from the manufacturing process	Residual reagents, ligands, and catalysts	Palladium, tungsten, lead, cadmium, arsenic, mercury, activated carbon	[7–9]
		Raw materials used, e.g., acids, alkalis, alkaline earth metal compounds.		
		Heavy metals, e.g., from process water, or leaching manufacturing equipment used such as reactors, micron filters, centrifuges and dryers.		
		Inorganic salts		
		Other materials, e.g., filtering aids, charcoal		

Residual solvents	Organic volatile chemicals used or produced during manufacturing that remain in the final product.	Residual solvents that are used as raw materials	Benzene, 1,2‐dichloroethane, carbon tetrachloride	[7, 10]
		They can also be produced during manufacturing		

As shown in Figure 1, organic impurities can originate from two main sources: the manufacturing process itself (process‐related) and the degradation of the drug over time (degradation‐related) [11, 12]. The levels of organic impurities are influenced by factors such as the synthesis route, purification methods, and stability of the API [3]. In response, global and national regulatory authorities have aligned their impurity control practices with the ICH guidelines. For instance, the Indonesian Pharmaceutical Regulatory Authority (BPOM) and the State Pharmacopeia of the Russian Federation XV adopt ICH frameworks such as Q3A (R2), Q3B (R2), and Q3C (R5) for impurity management [3].

**FIGURE 1:**
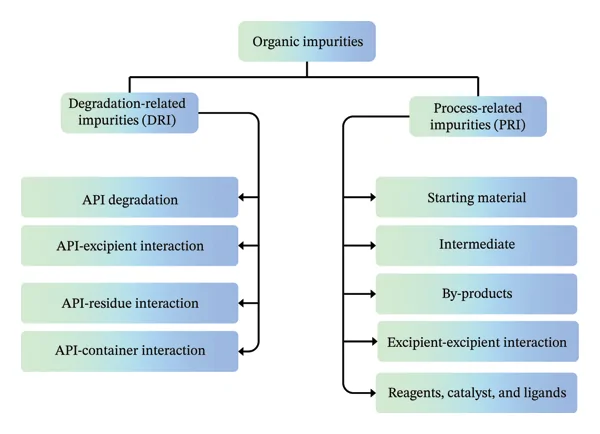
Sources of organic impurities.

A particularly critical subclass of organic impurities are genotoxic impurities (GTIs), which possess the potential to damage DNA leading to mutations, teratogenic effects, or carcinogenesis even at trace concentrations [13–16]. The presence of these GTIs at low concentration poses a significant analytical challenge, demanding the use of highly sensitive and selective techniques for their identification, control, and quantification [16, 17]. In response, regulatory bodies worldwide, including the European Medicines Agency (EMA), United States Food and Drug Administration (USFDA), and ICH, have established stringent guidelines, such as ICH Q3 and ICH M7, for the management of these hazardous substances throughout the drug lifecycle [2, 11, 18]. Within this category, hydrazine and its derivatives are of particular toxicological and regulatory concern due to their dual role as process‐related impurities and metabolically generated degradants in several pharmaceutical products.

As a process‐related impurity, hydrazine (N_2_H_4_) demonstrates the problems posed by the detection and management of GTIs in drugs. It is a colorless, ammonia‐smelling liquid belonging to a class of hydrazines, which are defined by two nitrogen atoms linked by a single covalent bond. Hydrazine is usually found in its stabilized form known as hydrazine hydrate (N_2_H_4_·H_2_O), as presented in Figure 2 [19, 20]. The compound has significant thermal instability and is highly reactive and corrosive. It is these characteristics, in addition to the high heat of combustion and reducing power which make hydrazine useful but difficult to handle [13, 21].

**FIGURE 2:**
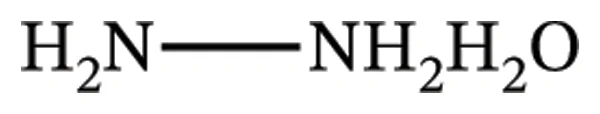
Structure of hydrazine hydrate.

The analytical detection of hydrazine is quite challenging because of its unique physical and chemical properties. Hydrazine has a low molecular weight, high polarity, and lack of chromophoric group and is chemically unstable, making direct analysis using routine chromatographic techniques difficult [20, 22, 23]. This has led to the development of sophisticated analytical methodologies, often involving chemical derivatization to introduce chromophoric groups that can form stable and detectable derivatives. In fact, various hyphenated techniques such as GC‐MS/MS, LC‐MS/MS, and UPLC coupled with fluorescence have been applied for trace‐level determination of hydrazine [11, 17, 20, 23–25]. Preanalysis sample treatment techniques, such as solid‐phase extraction (SPE) and liquid–liquid extraction (LLE), have generally been applied to preconcentrate hydrazine and minimize matrix interference [2, 22].

Most of the previous studies have largely focused on hydrazine’s toxicological risks, routes of formation, or detection techniques independently. This fragmented approach hinders a comprehensive understanding required for efficient impurity control. In order to fill this gap, this review combines toxicological properties, routes of formation of hydrazine during API synthesis, and recent advancements in extraction and analytical techniques into a single, practical framework covering developments from 2020 to 2025. This paper provides an applicable framework for impurity profiling as it brings all the elements together and enhances regulatory compliance efforts, with the ultimate goal of ensuring drug safety.

## 2. Hydrazine Impurity in Drug Substance

Hydrazine hydrate is widely employed as a synthetic reagent in the manufacturing of several APIs, including isoniazid [13, 22, 26, 27], allopurinol [18, 22, 26], hydralazine [13, 18, 22, 26], pantoprazole sodium sesquihydrate [28], carbidopa [13, 18, 26], 1‐hydroxybenzotriazole (HOBT) [15, 29], povidone [26], dihydralazine [26], nifuroxazide [13, 26], iproniazid [13], tecovirimat [30], nitrofural, nitrofurazone nitrofuroxazide, and pyridoxal isonicotinyl hydrazine [26]. Consequently, it can persist as a residual reagent or arise as a degradation product in the final drug product [13] such as isoniazid [31], hydralazine [32], ibrutinib [33], carvedilol phosphate [2], fluconazole, phenelzine, atazanavir [23], and avanafil [15]. This residual hydrazine may further react with synthetic intermediates, producing hydrazide impurities with potential mutagenic properties [15].

Hydrazine is classified as a Class 2 GTI under ICH M7 and a Group 2A carcinogen by the International Agency for Research on Cancer (IARC) indicating possible carcinogenic to humans [15, 17, 34, 35]. Class 2 impurities are defined as known mutagens with positive bacterial mutagenicity data but lacking rodent carcinogenicity studies, requiring control at or below the acceptable intake limits [11, 16, 17, 36]. Hydrazine toxicological profile is severe, with documented risks of hepatotoxicity, neurotoxicity, hematotoxicity, and genotoxicity [19, 31].

Strict threshold of toxicological concern (TTC) has been set by regulatory agencies in response to the serious toxicological risks caused by hydrazine. According to EMA, limits are set as low as 1.5 µg/day for long‐term exposure and 120 µg/day for short‐term treatments lasting less than a month [18, 37]. Overall, the acceptable limit for GTIs is generally low; this creates difficulties for controlling their presence during drug synthesis and process management [15]. Therefore, it is important to reliably detect, quantify, and evaluate the toxicity of hydrazine at all stages of drug manufacturing, formulation, and quality control to meet the strict regulatory requirements that have been set and ensure patient safety.

The possible synthetic routes for the formation of hydrazine and its related impurities during drug synthesis are shown in a schematic diagram in Figure 3.

**FIGURE 3:**
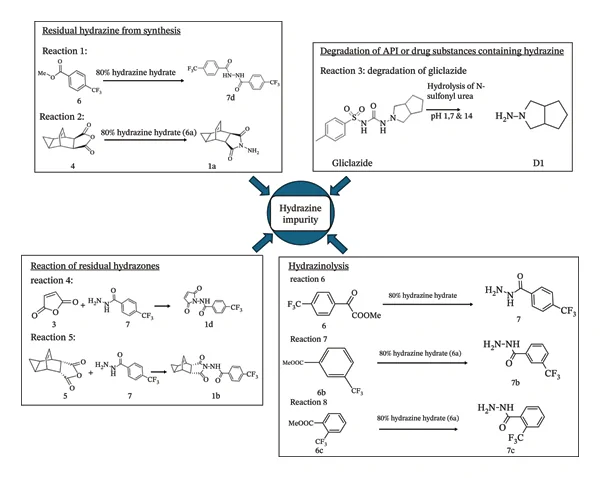
Synthesis pathways of hydrazine impurity in pharmaceutical substances.

Figure 3 highlights four synthesis pathways that contribute to the formation of hydrazine impurity and its derivatives, including the following:

1. Residual hydrazine from synthesis.
 Hydrazine or its salt, hydrazine hydrate, is a common reagent in the synthesis of APIs. If hydrazine is not completely removed during the purification stage, residual hydrazine can remain in the drug substance as an impurity or react with other compounds to form hydrazide‐related impurities.
 
*Reaction 1*: For instance, according to the scheme diagram during the synthesis of tecovirimat API, compound (6) can react with residual 80% hydrazine hydrate, leading to the formation of the impurity compound (7d).
 
*Reaction 2*: Intermediate compound (4) can also react with residual hydrazine (6a) to form hydrazine‐related impurity (1a) [30].
2. Degradation Products of APIs
 During storage, drugs that contain hydrazine moieties such as hydrazone or hydrazides can degrade due to moisture or light exposure.
 
*Reaction 3:* For example, gliclazide can degrade to form a hydrazine through the hydrolysis of its urea structure or by photolysis [38].
3. Reaction of residual hydrazones with other residual compounds.
 Residual hydrazones from the hydrazinolysis step can react with starting materials or other residual impurities, resulting in the formation of residual impurities.
 Reactions 4 and 5
 For example, in the manufacture of tecovirimat, residual hydrazone intermediate (7) reacts with residual compound (3) remaining in the Diels–Alder product and isomer by‐product compound (5) to form impurities 1d and 1b, respectively [30].
4. Formation from the hydrazinolysis step.
 Hydrazinolysis is the synthesis of hydrazides from carbonyl compounds and hydrazine. Hydrazine plays an important role in the synthesis of drug intermediates.
 
*Reaction 6*: For example, hydrobenzoyl/benzohydride, an intermediate formed from the hydrazinolysis of esters with hydrazine in the synthesis of tecovirimat API. The synthesis of tecovirimat API involves the hydrazinolysis step, where a methyl ester group in the benzoate moiety of compound 6 undergoes substitution with the hydrazide group from 80% hydrazine hydrate solution forming hydrobenzoyl/benzohydride intermediate compound (7).
 
*Reaction 7 and 8*: Side reactions can occur from the residual reactive methyl esters fragments compound (6b) and (6c) reacting with hydrazine hydrate compound (6a), resulting in the formation of in process impurities (7b) and (7c), respectively [30].

## 3. Toxicological of Hydrazine

Hydrazine, a process‐related impurity and a metabolic by‐product, leads to significant systemic exposure. It exhibits a well‐characterized profile of multiorgan toxicity, primarily targeting the central nervous system [19, 31], liver [27, 31], and blood [19], with confirmed genotoxicity and carcinogenic potentials summarized in Table 2 [2, 19, 27]. The clinical relevance of this is most clearly demonstrated by drugs like the antituberculosis agent isoniazid, which undergoes *in vivo* metabolism to release hydrazine and its derivatives; these metabolites are primarily responsible for the observed toxic effects in patients [27, 31, 34]. Systemic absorption has been confirmed in humans, where hydrazine has been detected in blood and plasma following the oral administration of isoniazid dosing (300 mg/day). This establishes a direct pharmacokinetic link between oral exposure and subsequent toxic manifestations [19]. This section describes the processes on how hydrazine damages the major organs after drug administration as shown in Table 2. It details the toxicological profile of hydrazine by elucidating the mechanisms by which it induces damage across these key organ systems following exposure from drug administration.

**TABLE 2:** Toxicology of hydrazine on specific target organs.

Target organ	Primary mechanism	Key toxic effects	Clinical manifestations	Ref.
Liver (hepatotoxicity)	Metabolic activation to reactive species (via NAT2/CYP2E1); oxidative stress and disrupted lipid metabolism.	Hepatocellular necrosis, fatty liver degeneration, and hyperammonemia.	Hepatotoxicity, elevated liver enzymes, liver failure	[19, 27, 31, 32]
Nervous system (neurotoxicity)	Pyridoxine (B6) antagonism ⟶ P5P ⟶ GABA synthesis ⟶ glutamate.	Neuronal hyperexcitability	Seizures, tremors, ataxia, restlessness, coma.	[19, 31]
Blood system (hematotoxicity)	Oxidative denaturation of hemoglobin	Hemolytic anemia, methemoglobinemia, Heinz body formation.	Fatigue, cyanosis, hemolysis.	[19]
Genome (genotoxicity)	DNA adduct formation, chromosomal breaks, mutations.	Direct damage to genetic material.	Potential initiation of cancer and other mutations.	[2, 27, 34, 35, 39]
Systemic (carcinogenicity)	Initiation of tumorigenesis via genotoxic mechanisms.	Development of benign and malignant tumors	Hepatocellular adenomas and carcinomas.	

### 3.1. Hepatotoxicity

Hydrazine induced hepatotoxicity results after its metabolism in the liver. The presence of a highly reactive –NH_2_ group in hydrazine and its derivatives facilitates the acetylation of hydrazine by N‐acetyltransferase enzyme type 2 to acetylhydrazine, which is further oxidized by cytochrome P450 enzymes (CYP2E1), to produce toxic metabolites [19, 31]. These metabolites cause oxidative stress in hepatocytes, depleting antioxidants such as superoxide dismutase (SSD) and glutathione peroxidase, which promotes lipid peroxidation and cell death [31]. The clinical significance of this metabolic pathway is revealed by studies carried out on hydralazine, a commonly used antihypertensive drug containing hydrazine moieties. This drug is known to have caused fulminant liver failure, which required urgent liver transplantation. Hepatotoxicity is believed to result from the metabolism of the drug into reactive and immunogenic compounds that trigger massive hepatocyte necrosis [32]. Hydrazine hepatoxicity is characterized by fatty liver, cell degeneration, and localized hepatic necrosis [19]. Meta analysis from clinical studies confirms that the risk of hepatotoxicity is higher in slow acetylators due to metabolite buildup compared to rapid acetylators [27, 31]. Recent preclinical studies continue to reinforce the hepatotoxic profile of hydrazine by demonstrating that hydrazine exposure produces reproducible clinical chemistry biomarker alterations that accurately predict the progression of liver toxicity [40].

### 3.2. Neurotoxicity

Hydrazine and its derivatives, particularly isoniazid metabolite INH‐hydrazine, are neurotoxic because they interfere with the production of gamma‐aminobutyric acid (GABA), a neurotransmitter inhibitor [19]. The highly nucleophilic–NH_2_ group at the end of INH reacts with pyridoxine (Vitamin B6), forming hydrazones [31]. These hydrazones inhibit the enzyme pyridoxine phosphokinase, which is responsible for the formation of pyridoxal‐5′‐phosphate (P5P). They also bind to the active P5P forming hydrazone complexes, which are eliminated via kidneys, effectively doubling the loss of Vitamin B6. This deficiency of P5P affects the catalysis of the glutamic acid decarboxylase enzyme, which is used to convert glutamate to GABA, leading to neuronal hyperexcitability [19]. Clinically, this mechanism manifests in both the central and peripheral nervous system. Central symptoms include tremors, restlessness, hyperreflexia, ataxia, confusion, refractory seizures, and coma [19], while peripheral neuropathy may present tendon reflexes, numbness, muscle pain, and weakness [31].

### 3.3. Hematotoxicity

Hydrazine also causes damage to red blood cells through the oxidative denaturation of hemoglobin, resulting in conditions such as hemolytic anemia, methemoglobinemia, and formation of Heinz bodies. This toxic consequence demonstrates hydrazine’s strong reducing properties, which weaken the stability of the red blood cell membranes and cause them to break down after oral exposure. These changes highlight the systematic toxicological effects of hydrazine on blood after oral drug administration [19].

### 3.4. Genotoxicity and Carcinogenicity

Hydrazine is classified as a Class 2 GTI under the ICH M7 guideline reflecting its DNA reactive and carcinogenic potential [15, 23, 34]. Hydrazine induces genotoxicity through multiple mechanisms including formation of DNA adducts, DNA methylation, chromosomal breaks, and mutations, which can initiate tumorigenesis [2, 27, 41]. Spencer and Kisby explained how hydrazine induces genotoxicity through DNA methylation and oxidative DNA damage, resulting in the formation of lesions such O^6^‐methylguanine, N^7^‐methylguanine, and 8‐oxo‐guanine, which promote mutations and uncontrolled cell division in cycling cells, which is a hallmark of cancer in postmitotic neurons [35]. In addition to DNA methylation, experimental evidence suggests that hydrazine‐containing compounds may also induce genotoxicity through direct DNA modification. Hydralazine drug has been shown to form stable covalent adducts with apurinic/apyrimidinic (AP) sites in DNA, providing an additional pathway through which hydrazine‐containing compounds may compromise genomic instability [41]. In long‐term animal studies, exposure to hydrazine resulted in a statistically significant increase in hepatocellular adenomas and carcinomas upon exposure validating its carcinogenic classification [27].

Toxicological studies consistently reaffirm that hydrazine exposure, whether as a process residue, degradation product, or through metabolic exposure, presents significant neurotoxic, hepatotoxic, and genotoxic risk, supporting the current ICH M7 classification as a Class 2 GTI [19, 27, 31, 32].

## 4. Extraction and Sample Preparation of Hydrazine

The detection of hydrazine is technically challenging because of its low molecular weight, high polarity, strong reductivity, lack of chromophore, and unstable physical and chemical properties [20, 22, 23]. To address these challenges, as outlined in Table 3, most recent analytical work flows for hydrazine employ sample preparation techniques that merge chemical derivatization and extraction prior to instrumental analysis, thereby improving stability and detectability [2, 20, 22, 33].

**TABLE 3:** Sample preparation and analytical methods for the determination of hydrazine impurity in drugs.

No.	Matrix/API	Impurity	Extraction technique	Derivatization reagent	Analytical method	System	Results (ppm)^a^	Ref.
1	Carvedilol phosphate drug	Hydrazine, phenyl hydrazine	Liquid–liquid extraction (LLE) using t‐butyl methyl ether (MTBE) after dissolution in N‐methyl‐2‐pyrrolidone (NMP)	Acetone	GC‐MS	Column‐DB—5 ms, 30 m length i.d.: 0.32 mm, film thickness—1.0 µm Stationary phase—(5%‐phenyl)‐methylpolysiloxane Injection volume—1 µl Split ratio—1:15 Temperature—40°C, 30°C, 210°C, 300°C Carrier gas—helium gas Flow rate—1.5 mL/min Dwell time—6–12 cycles) Ionizing energy—70 eV Ion type—electron ionization mode with selective ion monitoring (SIM) Detector—MS	LOD—0.06–0.61 ppm Split ratio—1:15	[2]

2	Pantoprazole sodium sesquihydrate	Hydrazine	—	Salicylaldehyde	RP‐HPLC	Column—Inertsil ODS‐3V (250 × 4.6 mm, particle size 5 µm). Temperature: 30°C Mobile phase: ammonium dihydrogen phosphate buffer: methanol (25:75 v/v) Isocratic Detector—PDA	LOD—1.03 ppm LOQ—3.1 ppm	[28]

3	Prednisolone	Hydrazine	—	Acetone	GC‐MS/MS	GC oven temperature: ±5°C Heating rate: ±2° C/min Column flowrate: ±0.1 mL/min Column—VF‐5 MS capillary column (30 m × 0.25 mm *x* 0.25 µm) Flowrate: 1.0 mL/min Injection port temperature: 250°C Carrier gas: helium Split injection at ratio: 5:1 Injection volume—1.0 µL Constant linear velocity control mode MS/MS conditions: electron impact ion source (EI) Electron energy: 70 eV Ion source temperature: 230°C Interface temperature: 280°C Solvent delay time: 2 min Multiple reaction mode (MRM)	LOD—0.03 ppm LOQ—0.10 ppm	[20]

4	Ibrutinib drug	Hydrazine hydrate	—	Salicylaldehyde	RP‐HPLC	Mobile phase: ammonium dihydrogen phosphate buffer (0.02 M) and Acetonitrile in the ratio of 35:65 v/v. Stationary phase: Octadecyl Isocratic elution Column: Waters X ‐Terra RP18 250 mm × 4.6 mm *x* 5.0 µm. Stationary phase: Octadecyl Isocratic elution Gradient pump Column temperature: 40°C Injection volume: 20 µL Detector—UV wavelength: 354 nm Flow rate: 1.0 ml/min	LOD—0.27 ppm LOQ—0.81 ppm	[33]

5	Allopurinol	Hydrazine	—	O‐phthalaldehyde (OPA)	UHPLC‐fluorescence	Reverse phase Binary gradient elution. Mobile phase: A‐20 mM phosphate buffer (pH 2), B‐acetonitrile. Flowrate: 0.5 mL/min Injection volume: 5 µL Column (C 18 BEH): 50 × 2.1 mm, 1.7 µm Column temperature: 30°C Detector: fluorescence (315/370 nm) and PDA	LOD—0.03 ppm. LOQ—0.25 ppm	[18]
6	Allopurinol	Hydrazine	Online‐solid‐phase extraction (SPE) using hydrophilic–lipophilic technology sorbent.	O‐phthalaldehyde (OPA)	Zone Fluidics, HPLC (corroborative studies)	Microelectrically actuated 10‐port valve (Valco) and a minipulse (Gilson) peristaltic pump Holding coil (HC)—300 cm × 0.7 mm i.d. Stainless steel rod—4.6 mm i.d. Injection volume (buffer and derivatizing reagent) —50 µL Sample volume—200 µL. Flowrate—0.6 mLmin^−1^ Reaction coil (RC)‐ 100 cm × 0.5 mm i.d. Detector—RF‐551 spectrofluorimetric (*ƛ* = 318/376) Fluorescence spectra recorded using RF‐5301PC batch spectrofluorophotometer.	LOQ—2.7 ppm LOD—0.9 ppm	[22]

7	Gliclazide	Hydrazine	—	3,4,5‐Trimethoxy benzaldehyde	UPLC	Column: YMC Pack C18 (50 × 3.0 mm i.d., 2 µm) Oven temperature: 40°C. Flowrate: 0.6 ml/min Gradient elution Mobile Phase A: 0.1% Trifluoroacetic acid Mobile Phase B: acetonitrile Detection: UV, 333 nm	LOD—0.10 ppm LOQ—0.30 ppm	[25]

8	Argatroban monohydrate	Hydrazine	—	—	Chiral HPLC‐UV	Column: YMC chiralart cellulose‐SC, 250 × 4.6 mm *x* 5 µm Normal phase Linear elution Mobile phase: methanol: acetonitrile: dichloromethane: trifluoroacetic acid: triethylamine (30:50:920:3:4) Flowrate: 0.7 mL/min Detector: UV/PDA (259 nm) Injection volume: 5 µL Column oven temperature: 35°C	LOD‐30^b^ ppm LOQ‐100^b^ ppm	[42]

9	Ondansetron hydrochloride	Phenyl hydrazine	—	—	LC‐MS/MS (UPLC‐ H‐class system)	Column—C18, 5 µ (150x 4.6) mm. Flowrate—0.6 ml/min Mobile phase—5 mM ammonium formate as mobile phase A, acetonitrile as Mobile Phase B. Column temperature—35°C Injection volume—20 µL Elution—gradient Detector—MS (electrospray ionization in positive mode). Capillary voltage—3 KV MMR mode. Collision energy −20 V, cone voltage—15 V, desolvation gas flow—900 L/hr with a temperature of 450°C and source temperature at 150°C	LOD—5 ppm LOQ—15 ppm	[24]

10	Apixaban drug	Ethyl (Z)‐2‐chloro‐2‐(2‐phenyl hydrazinylidene) ethanoate, ethyl(Z)‐2‐chloro‐2‐(2‐[‐methoxyphenyl]hydrazineylidene)acetate, ethyl(Z)‐2‐chloro‐2‐(2‐[4‐chlorophenyl]hydrazineylidene)acetate, ethyl(Z)‐2‐chloro‐2‐(2‐[2‐methoxyphenyl]hydrazineylidene)acetate.	—	—	UPLC‐UV	Column: ACE excel C18‐PFP (100 mm × 2.1 mm, 1.7 µm) Column oven temperature: 45°C Mobile Phase A: 0.1% orthophosphoric acid in water Mobile Phase B: 100% acetonitrile Flowrate: 0.2 ml/min Detection wavelength: 330 nm Injection volume: 2.0 µL	LOD: 3.6 ppm LOQ: 12.0–12.2 ppm	[43]

^a^Concentrations originally reported as in different units were standardized to parts per million (1 ppm = 10^−6^) to allow direct comparison across all studies. Values originally reported on a mass/mass basis (mg kg^−1^, µg g^−1^) are unchanged, since 1 µg g^−1^  = 1 mg kg^−1^  = 1 ppm. Values originally reported as % (w/w) were converted using ppm = % × 10^4^. Values originally reported on a mass/volume basis (mg kg^−1^, mg mL^−1^) were first placed on a mass basis by assuming a solution density of 1.00 g mL.^−1^ (i.e., 1 ml of solution ≈ 1 g), the dilute‐solution assumption adopted for the aqueous solutions by Korbag et al. 2022, on the basis 1 µg mL.^−1^  = 1 ppm and 1 mg mL.^−1^  = 1000 ppm. Conversion relations follow Korbag et al. 2022 [44].

^b^The reported LOD (30 ppm) and the LOQ (100 ppm) are relatively high and may not provide sufficient sensitivity for the current regulatory control of a mutagenic impurity under ICH M7.

### 4.1. Sample Preparation

Sample preparation represents a critical step in the determination of hydrazine‐related impurities because analyte losses can occur before instrumental analysis. Adsorption of (4‐sulfamoylphenyl)hydrazine (4‐SAPH) to glass surfaces during celecoxib sample preparation has been shown to reduce analyte recovery. The use of polypropylene containers and ammonium hydroxide effectively minimized adsorption and improved quantification accuracy [45].

### 4.2. Derivatization

In the reviewed literature summarized in Table 3, all methods except Dhalape et al. (2020) [42], Kotapati Nalini et al. (2020) [24], and Ganta et al. (2024) [43] employed a derivatization step prior to analysis. Chemical derivatization is a technique that is used to modify the structure of the analyte into a new chemical compound (derivative) through a specific chemical reaction [2, 18, 20, 25, 28, 33]. This step is essential for converting hydrazine into a more stable and readily detectable derivative by introducing chromophoric groups [16, 22]. The resulting chemical transformation typically yields products with reduced polarity and increased molecular weight, thereby enhancing compatibility with chromatographic systems. Beyond improving chromatographic behavior, derivatization also increases fluorescence, induces a bathochromic shift, enabling detection at higher wavelengths, enhances ionization efficiency for mass spectrometry, and increases thermal volatility for GC [16, 28, 46]. As noted by T. Krishnamohan et al. (2022) [28], this spectral shift facilitates detection in regions where API components exhibit minimal absorbance, thereby improving analytical specificity and reducing matrix interference.

For Entries 2, 4, and 7 in Table 3, PDA/UV detection is enabled by the formation of a chromophoric hydrazone through the reaction of hydrazine with an aldehyde reagent [25, 28, 33]. Thus, the UV response arises from the derivatized hydrazone rather than from the direct detection of unmodified hydrazine, which lacks a suitable chromophore.

As outlined in Table 3, the choice of derivatizing agent varies by analytical technique. Acetone forms stable hydrazones suitable for high temperature of gas chromatography (GC‐MS, GC‐MS/MS) as seen in carvedilol and prednisolone methods [2, 20]. In liquid chromatography (HPLC, UHPLC)–based methods, salicylaldehyde and *o*‐phthalaldehyde (OPA) are commonly used forming fluorescent or UV‐absorbing derivatives as seen in the analysis of carvedilol phosphate drug [2], pantoprazole sodium sesquihydrate [28], and allopurinol [18]. For ultraperformance liquid chromatography (UPLC), 3,4,5‐trimethoxybenzaldehyde is the preferred agent in the analysis of gliclazide [25], while OPA is also reported for flow analysis techniques in the analysis of allopurinol [22].

### 4.3. Extraction Techniques

The effective isolation of hydrazine or its derivatives from complex pharmaceutical matrices often necessitates extraction techniques to preconcentrate the analyte and reduce the matrix interference. Several extraction strategies, such as SPE [22],LLE [2], have been developed to isolate hydrazine from pharmaceutical matrices.

#### 4.3.1. LLE

LLE, also known as solvent or partitioning extraction, is a separation technique that partitions an analyte between two immiscible liquids, typically an aqueous phase and an organic solvent, based on its differential solubility [47]. This principle is demonstrated in the work for the analysis of hydrazine and phenylhydrazine in carvedilol phosphate [2]. The method involved derivatizing hydrazine and phenylhydrazine with acetone, followed by LLE with t‐butyl methyl ether (MTBE) as the organic solvent. This step quantitatively transferred the stable derivatives from the aqueous sample into the organic layer, ensuring efficient phase separation.

This approach achieved good sensitivity (limit of detection [LOD]—0.06 ppm) and LOQ (0.17 ppm) with a wide but acceptable recovery range of 85.3%–109.5% while minimizing matrix interference during subsequent using GC‐MS analysis [2]. However, conventional LLE is a manual process, involves multisteps that consumes significant volume of organic solvent, and is less suitable for high‐throughput automation [47].

#### 4.3.2. SPE

In contrast, SPE offers a more modern alternative. It is a powerful sample preparation technique that isolates and concentrates analytes from complex matrices by distributing them between a liquid phase (sample medium) and a solid phase (adsorbent), allowing for selective retention and purification. Generally, the target analyte demonstrates a stronger affinity for the solid sorbent material and is selectively retained as the sample solution flows through the extraction medium [48]. This extraction technique is highly effective for isolating and concentrating challenging compounds such as hydrazine and its derivatives as it removes matrix interferences [22]. Moreover, SPE stands out for reducing organic solvent consumption and its compatibility with automated workflows which minimize manual handling and improve consistency across runs [48].

A practical demonstration of these advantages is shown in the study by Paraskevas et al. (2020), which integrated online SPE using hydrophilic–lipophilic balance (HLB) sorbents into a zone fluidics setup for the determination of hydrazine in allopurinol [22]. Although the method achieved acceptable analytical performance, with LOQ (2.7 ppm) and recoveries between 93.3% and 105.8%, its LOD of 0.9 ppm was significantly higher than the LLE/GC‐MS methods (0.06 ppm). The sensitivity was limited by the type of fluorometric detector being utilized, rather than the extraction step itself. Overall, this work exemplifies how SPE can function as a powerful method to increase both reproducibility and productivity in those cases where the detector’s sensitivity is already sufficient for the required analytical threshold.

More recently, greener and miniaturized extraction techniques including solid‐phase microextraction (SPME), stir‐bar sorptive extraction (SBSE), microextraction by packed sorbent (MEPS), fabric phase sorptive extraction (FPSE), and solvent‐based microextraction (SBME) have gained increasing attention as alternatives to conventional extraction approaches [49, 50]. However, within the 2020–2025 literature reviewed for pharmaceutical drug matrices, no clear application of such techniques specifically for hydrazine determination was identified. Therefore, LLE and SPE remain the principal extraction approaches reported for hydrazine analysis in pharmaceutical matrices within the scope of this review, while the application of greener and miniaturized extraction strategies represents the potential area for future methodological development.

In some cases, direct preparation without extraction is sufficient, provided that an appropriate derivatizing agent is used and the API matrix is not very complicated. For instance, salicylaldehyde was used as a derivatizing agent during the analysis of hydrazine hydrate in ibrutinib using RP‐HPLC, achieving LOD 0.27 ppm and LOQ 0.81 ppm, with high recoveries greater than 99.3% [33]. Another study investigated the determination of hydrazine derivatives in gliclazide using 3,4,5‐trimethoxybenzaldehyde as a derivatizing agent [25]. A stable hydrazone with strong UV absorbance at 333 nm yielded an LOD of 0.10 ppm, LOQ of 0.30 ppm, and recoveries greater than 96.9% [25].

While many hydrazine assays rely on derivatization to enhance detection, some LC‐MS/MS methods, such as the one reported for phenyl hydrazine in ondansetron HCl, employ direct dilution of API and analysis without extraction or derivatization steps [24]. By leveraging the high selectivity and sensitivity of tandem MS, it achieved a respectable LOD of 5 ppm, LOQ of 15 ppm with good recovery range (93.6%–112.3%) [24]. This workflow significantly reduced sample handling while achieving trace detection. Conversely, the nonderivatized chiral HPLC‐UV method for Argatroban, described by Dhalape et al. (2020), is the worst‐case scenario for this approach [42]. The method is grossly inadequate with its LOD of 30 ppm, LOQ of 100 ppm, being completely substandard by contemporary standard concerning GTIs, although with decent recoveries ranging 100%–103.2% [42]. This serves as a clear warning that without either derivatization or a highly selective detector, achieving the necessary trace‐level sensitivity is very unlikely.

Handling complex matrices is one of the greatest challenges in hydrazine analysis. Therefore, techniques like LLE and SPE are still essential for concentrating and reducing matrix interference in order to improve method performance and recovery rates [2, 22]. However, the reviewed literature shows that no single extraction method is supreme for hydrazine analysis; instead, the choice depends on the requirements of the analysis. LLE combined with mass spectrometry is the most reliable method for achieving high sensitivity (LOD < 0.1 ppm) to meet strict GTI limits [2]. On the other hand, SPE is more compatible with automated, high‐throughput workflows in laboratories that prioritize speed and consistency despite its slightly higher LOD values (~ 1 ppm). Zone fluidics automation shows potential, although its sensitivity is still moderate with LODs around 0.9 ppm [22]. Factors such as matrix complexity, derivatization reagent used, and analytical instrumentation available influence method selection. Apart from their differences, all methods aim to stabilize hydrazine, minimize analytical interference, and accurately measure trace‐level concentrations in compliance with ICH M7 (R2).

## 5. Comparative Evaluation of Analytical Methodologies: Derivatization and Detection Strategies

The comprehensive literature review summarized in Table 3 reveals that a range of sensitive and selective methods such as liquid chromatography (HPLC [28, 33, 42], UHPLC [18], UPLC [25]), flow analysis (zone fluidics) [22], and hyphenated techniques (GC‐MS [2], GC‐MS/MS [20], and LC‐MS/MS [24]) have been developed for detecting hydrazine and its derivates in APIs and finished drug products. Hyphenated techniques are crucial for the identification and measurement of GTIs like hydrazine. These techniques combine chromatography, which separates the complex mixtures into individual components, and spectroscopy, which identifies each component by matching it against the reference standards or spectral libraries [13].

All the reported LODs and LOQs presented in Table 3 were converted to the same unit ppm for ease of comparison. This uniformity enabled the assessment of analytical sensitivity across spectrophotometric, chromatographic, and flow‐based methods.

The results revealed a broad range of detection sensitivities for hydrazine, mostly dependent on the extraction method, derivatizing reagent, analytical technique, and detection system used. Assessment of these analytical methods shows the distinct patterns in terms of sensitivity, accuracy, and precision.

### 5.1. The Supreme Sensitivity of Tandem Mass Spectrometry and Fluorescence Detection

Evidently, derivatization‐based methods coupled with advanced detection are the most sensitive techniques. The analysis of hydrazine in prednisolone using GC‐MS/MS [20] and in allopurinol using UHPLC‐fluorescence [18] achieved extremely high sensitivity with the same LOD as low as 0.03 ppm. Both methods presented outstanding accuracy with percentage recovery ranges of 96.15%–96.46% and 92.7%–106.7%, respectively. These findings show the power of the GC‐MS/MS [20] while also highlighting that fluorescence detection can serve as an alternative, as it is more accessible and is highly sensitive when combined with an appropriate derivatizing reagent. However, the two methods differ significantly in precision, where the GC‐MS/MS method achieved excellent repeatability showing very low interday variability (RSD of 1.77%) [20], while the UHPLC‐fluorescence [18] method demonstrated higher but still acceptable, interday variability (RSD ≤ 6.9%). These results clearly indicate that the GC‐MS/MS method may achieve greater robustness for routine analysis compared to the UHPLC‐fluorescence method.

Likewise, the GC‐MS method for the analysis of hydrazine in carvedilol phosphate also offers high sensitivity in the sub‐ppm level, achieving an LOD of 0.06 ppm with a broad but acceptable recovery range of 85.3%–109.5%, even though its precision demonstrated a wide RSD range between 0.1% and 4.7% [2]. Prednisolone GC‐MS/MS [20] method exhibits a very low LOD of 0.03 ppm compared to that of the standard GC‐MS [2] for carvedilol phosphate with an LOD of 0.06 ppm, even though both methods used acetone as their derivatization reagent. This demonstrates the significant benefit of adding the second mass analyzer (MS/MS).

### 5.2. High Performance From Modern Chromatography With Derivatization

Chromatographic techniques also showed high sensitivity as illustrated by the method for the determination of hydrazine in gliclazide using UPLC [25] and ibrutinib using RP‐HPLC [33], which achieved impressive LODs of 0.10 ppm and 0.27 ppm, respectively. These methods not only showed high sensitivity but also achieved excellent accuracy with recoveries falling between 96.9% and 106.8% and significantly low precision with RSDs less than 0.62%. These observations demonstrate that modern chromatographic techniques are highly suitable for the analysis of Hydrazine when combined with effective derivatization reagents, offering robustness together with high sensitivity.

In cases where extreme sensitivity is not a primary requirement, other methods such as RP‐HPLC for pantoprazole [28] and automated zone fluidics for allopurinol [22] offer medium sensitivity with valuable advantages of simplicity and automation.

The derivatized RP‐HPLC method for pantoprazole fulfilled all validation requirements by achieving an LOD of 1.03 ppm with good recovery of 97.80%–100.18% and precision RSD of 2.49% [28]. However, its LOD of 1.03 ppm is significantly higher compared to other derivatized HPLC methods such as ibrutinib and gliclazide with LODs of 0.27 ppm [33] and 0.10 ppm [25], respectively. This difference highlights that the API matrix and the choice of derivatizing agent can influence the final sensitivity, although the same analytical technique is used.

Meanwhile, the analysis of hydrazine in allopurinol using automated zone fluidics offers an advantage of full automation with an acceptable LOD of 0.9 ppm and good recovery in the range 93%–105.8%, although it demonstrated the highest interday precision with RSD (≤ 8.6%) in the dataset [22].

### 5.3. The Underivatized Compromise: How Detection Defines the Trade‐Off

The underivatized ondansetron hydrochloride LC‐MS/MS method, presented by Palnati et al. (2020), achieved a lower LOD of 5 ppm with good recovery in the range 93%–112.3% and precision with RSD of 1.2% compared to the underivatized Argatroban Chiral HPLC method reported by Dhalape et al. (2020), which reported a significantly high LOD of 30 ppm despite its good recovery values between 100% and 103% and precision between 1.08% and 1.70% [24, 42]. This illustrates that the elevated selectivity and sensitivity of the tandem mass spectrometry in underivatized LC‐MS/MS method can to some extent compensate for the lack of a derivative.

However, both methods [24, 42] exhibited the poorest sensitivity compared to the rest of the methods in Table 3 with significantly high LODs, highlighting the importance of derivatization despite the high sensitivity of the instrument. The underivatized Argatroban Chiral HPLC‐UV method was a clear outlier exhibiting the poorest sensitivity among all the methods discussed, with an extremely high LOD of 30 ppm [42]. These two methods reveal the compromise between convenience and sensitivity that in as much as excluding the derivatization step simplifies sample preparation, it can result in significant loss of sensitivity, especially when using conventional detectors such as UV–vis. Such methods can only be used if the intended application can tolerate higher detection limits.

The level of sensitivity follows a clear hierarchy directly related to the detection system used. The derivatized hyphenated technique, particularly the GC‐MS/MS [20], achieves the same lowest LOD of 0.03 ppm with the derivatized UHPLC‐fluorescence [18]. This makes both methods benchmark for compliance with the stringent regulatory limits. Although both techniques show equivalent supreme sensitivity, examining the rest of the validation parameters uncovers distinct operational advantages. The GC‐MS/MS method for the analysis of hydrazine in prednisolone demonstrates outstanding precision, with an interday RSD of 1.77% with good recovery values closely grouped between 96.15% and 96.46% [20]. In contrast, the UHPLC‐fluorescence shows more inconsistent interday RSD ≤ 6.9% with a very wide recovery range of 92.7%–106.7% [18].

This highlights that GC‐MS/MS offers greater robustness and precision compared to UHPLC‐fluorescence, which is a key benefit for routine quality control applications where method reproducibility is a priority. The excellent precision achieved by the GC‐MS/MS [20] over UHPLC‐fluorescence [18] and its superior performance over the standard GC‐MS [2] method, although both methods used the same derivatizing agent, acetone, can be associated with the second mass analyzer (MS/MS). The second mass analyzer enhances the selectivity by effectively eliminating coeluting matrix interferences that might contribute to the variability in fluorescence detection and the standard GC‐MS/MS methods.

The compiled data in Table 3 clearly show that methods that exclude derivatization incur significant loss in sensitivity, even though they are simpler and faster. This illustrates the compromise between analytical simplicity and sensitivity. The underivatized LC‐MS/MS method for Ondansetron with an LOD of 5 ppm reveals that the high selectivity of tandem mass spectrometry can partly compensate for the lack of derivative [24]. Therefore, it can be used as a possible viable alternative method for certain applications, though less sensitive. In contrast, the underivatized chiral HPLC‐UV method for Argatroban reported by Dhalape et al. (2020), which achieved an LOD of 30 ppm, is practically ineffective for determining GTIs [42]. Derivatization remains a vital step for reliable quantification of hydrazine, as instrumentation alone is not enough to compensate for its intrinsic instability and absence of the chromophoric group. However, the introduction of this additional step may compromise the consistency of results.

Toxicological risks, formation pathways, sample preparation ways, and advanced analytical techniques for hydrazine from 2020 to 2025 have been merged in this review in order to address the critical gap in the literature, whereby these aspects are often addressed independently. The extensive use of hydrazine during the manufacturing of APIs compels the need for this integrated insight.

The combination of toxicological insights and advances in analytical techniques has caused a paradigm shift toward using highly sensitive, derivatization‐based, and hyphenated chromatographic methods that can quantify analytes in trace concentrations. Assessment of analytical methodologies unveils that the primary challenge in the analysis of hydrazine lies on its unfavorable physicochemical properties such as high polarity, low molecular weight, lack of chromophore, and chemical instability. This is illustrated in Table 3, where most of the methods overwhelmingly addressed these issues through the application of chemical derivatization. Chemical derivatization is not just an optional improvement but an essential requirement that modifies hydrazine into a more stable, detectable, high‐molecular‐weight derivative exhibiting optimal chromatographic performance.

Chromatographic techniques such as UPLC [25] and RP‐HPLC [33], combined with effective derivatizing agents such as 3,4,5‐trimethoxybenzaldehyde or salicylaldehyde, respectively, can be used by quality control laboratories that may not have routine access to MS instrumentation. Such techniques demonstrate good sensitivity with LODs ranging between 0.10 and 0.27 ppm, moderate accuracy in the range of 93.6%–112.3%, and exceptional precision with RSD ≤ 0.62% [25, 33]. These findings establish them as highly reliable, robust, and more accessible cornerstone techniques for quality control testing.

## 6. Analytical Method Development, Optimization, and Validation

The comparison of the analytical methods discussed above demonstrates that hydrazine method development involves the selection and optimization of sample preparation, derivatization, chromatographic separation, and detection conditions. Differences in reported LOD, LOQ, recovery, and precision provide useful information on how these analytical factors can impact method performance. Thus, the reviewed studies can be considered not only in terms of their reported analytical performance but also in terms of the approaches used to develop and optimize the analytical procedures.

### 6.1. Optimization

The optimization of critical analytical parameters is an important component of method development. Christofi et al. 2020 provided an example of systematic univariate optimization of critical derivatization parameters and chromatographic conditions. The derivatization conditions were optimized by individually investigating OPA concentration, reaction time, pH, and temperature. A reaction time of 5 min and OPA concentration of 25 mmol/L were selected based on the response obtained, while pH 2 was selected as a compromise between fluorescence response and column stability. Chromatographic conditions were then optimized using allopurinol samples spiked at the specification limit. Buffer concentration, mobile‐phase composition, and column temperature were evaluated with 2% acetonitrile (ACN) and 30°C selected, and the resulting separation provided a resolution of 4.7 [18].

### 6.2. Analytical Quality by Design (AQbD)

The transition from one factor at a time (OFAT) or trial‐and‐error (TAE) method development to AQbD framework is now a strategic necessity. The core of the AQbD framework is the principle that analytical quality must be built‐in rather than tested‐in. It provides a systematic science and risk‐based approach in which the intended purpose and performance of the analytical procedure are defined before development, followed by identification and investigation of factors that may influence method performance. A typical AQbD approach may include the definition of an analytical target profile (ATP), risk assessment to identify potentially influential analytical parameters, systematic experimental investigation of these parameters, and establishment of an appropriate operation region [51, 52]. Design of experiments (DoE) is one of the statistical tools that can be applied within this framework because it enables multiple factors and their effects on analytical responses to be investigated systematically rather than evaluating each factor independently [18, 51].

The literature reviewed in this study shows that analytical method development factors have been addressed using conventional method development approaches as well as more structured experimental strategies. Therefore, the reported hydrazine methods can also be considered from the perspective of AQbD, while recognizing that the published methods do not necessarily represent the complete formal AQbD implementations.

The relevance of structured experimental approaches to hydrazine analysis has been demonstrated by Christofi et al. 2020 who constructed experimental designs to evaluate the robustness of both the instrument method and derivatization procedure. The robustness of the developed procedure was subsequently assessed using DoE. A Plackett–Burman design evaluated five chromatographic factors, with resolution, retention, peak asymmetry, and theoretical plates used as responses. Buffer pH and column temperature significantly affected resolution, while the worst‐case resolution remained 3.5. A separate fractional factorial design (FFD) with resolution IV was applied to assess the robustness of four derivatization factors, and no significant effects were observed; the peak area % RSD was 2.8%. The experimental results were evaluated using analysis of variance (ANOVA), with statistical significance assessed at p < 0.05 [18]. Similarly, Nakka et al. 2023 applied DoE to the robustness study of a UPLC‐derivatization method for hydrazine determination in gliclazide [25]. These examples illustrate that DoE can provide a systematic means of identifying influential analytical parameters and assessing method robustness. However, DoE is a tool within the broader AQbD framework rather than AQbD itself.

### 6.3. Analytical Method Validation

Following development, the analytical procedure should be validated. Validation provides evidence that the selected analytical procedure is fit for its intended purpose [18, 53]. The reported LOD, LOQ, recovery, precision, linearity, specificity, and robustness data provide complementary support for analytical performance rather than isolated characteristics. In accordance with ICH Q2(R2), analytical procedure validation forms part of the analytical procedure lifecycle and serves to prove that the procedure is fit for its intended purpose [53]. ICH Q2 (R2) is closely linked with ICH Q14, which addresses analytical procedure development and includes science and risk‐based approaches to development [51, 53]. Accordingly, method development, optimization, robustness assessment, and validation can be considered interrelated steps in establishing a reliable analytical procedure for hydrazine determination.

## 7. Conclusion

Hydrazine continues to be recognized as one of the most difficult and hazardous impurity to detect in the pharmaceutical industry due to its high volatility, reactivity, polarity, low molecular weight, and lack of chromophoric group. Its problematic physicochemical properties require the application of derivatization coupled with highly sensitive analysis techniques. Hydrazine’s toxicological concern is primarily linked to its metabolic conversion into reactive intermediates, supporting the need for trace‐level quantification and strict regulatory control. The overall findings from 2020 to 2025 reveal that derivatization‐based hyphenated techniques, specifically GC‐MS/MS, demonstrate superior sensitivity, precision, and robustness for trace‐level quantification, making it the gold analytical method. Alternative techniques such as UHPLC‐fluorescence [18] continue to improve accessibility as they adhere well to ICH and EMA GTI regulatory requirements.

Regardless of these advances, gaps still remain in method standardization, availability of reference standards, and validation of complex compositions.

Future research should focus on integrating, automated, green advanced analytical techniques with extensive toxicological assessment to ensure continuous pharmaceutical safety and adherence to evolving global regulations.

## Funding

This article has been funded with the support of the Kemitraan Negara Berkembang (KNB) Scholarship from the Ministry of Higher Education, Sciences, and Technology of the Republic of Indonesia on behalf of the Government of the Republic of Indonesia.

## Disclosure

This study reflects the view only of the author, and the Ministry of Higher Education, Sciences, and Technology of the Republic of Indonesia cannot be held responsible for any use, which may be made of the information contained therein.

## Conflicts of Interest

The authors declare no conflicts of interest.

## Data Availability

Data sharing is not applicable to this article as no datasets were generated or analyzed during the current study.
